# A prospective web-based patient-centred interactive study of long-term disabilities, disabilities perception and health-related quality of life in patients with multiple sclerosis in The Netherlands: the Dutch Multiple Sclerosis Study protocol

**DOI:** 10.1186/s12883-015-0379-0

**Published:** 2015-08-04

**Authors:** Peter Joseph Jongen, Marco Heerings, Wim A. Lemmens, Rogier Donders, Anneke van der Zande, Esther van Noort, Anton Kool

**Affiliations:** Department of Community & Occupational Medicine, University Medical Centre Groningen, Antonius Deusinglaan 1, 9713 AV Groningen, The Netherlands; MS4 Research Institute, Ubbergseweg 34, 6522 KJ Nijmegen, The Netherlands; MH-Advies & organisatiebureau, IJselstraat 81, 9406 TR Assen, The Netherlands; National MS Foundation The Netherlands, Mathenesserlaan 378, 3023 HB Rotterdam, The Netherlands; Department for Health Evidence, Radboud University Nijmegen Medical Centre, P.O. Box 9101, 6500 HB Nijmegen, The Netherlands; Curavista bv, Markt 9, 4931 BR Geertruidenberg, The Netherlands

**Keywords:** Multiple sclerosis, Clinically isolated syndrome, Long-term, Disability, Quality of life, Patient-driven, Patient-centred, Patient-reported outcomes, Web-based, Online

## Abstract

**Background:**

In the past two decades the widespread use of disease modifying drugs with moderate to strong efficacy has changed the natural course of multiple sclerosis (MS). Health care professionals, researchers, patient organizations and health authorities are in need of recent information about the objectified and subjective long-term clinical outcomes in MS patients. Such information is scarce.

**Methods/Design:**

We started a prospective, web-based, patient-centred, interactive study of long-term disabilities, disabilities perception and health-related quality of life (HRQoL) in MS patients in The Netherlands (Dutch Multiple Sclerosis Study). The study has an on online patient-driven inclusion and online acquisition of patient-reported outcomes (PROs). At six-months intervals participants complete the Multiple Sclerosis Impact Profile (MSIP) (disabilities and disabilities perception in seven domains and four symptoms), the Multiple Sclerosis Quality of Life-54 items (MSQoL-54), the Modified Fatigue Impact Scale-5 items (MFIS-5) and the Leeds Multiple Sclerosis Quality of Life-8 items (LMSQoL) questionnaires, and a Medication and Adherence Inventory. Every three years the Expanded Disability Status Scale (EDSS) score is assessed by phone. The monthly completion of the MFIS-5, LMSQoL and Medication and Adherence Inventory is optional. Completed questionnaires and inventories, and automatically generated scores are made available online to patients for self-monitoring and self-management purposes, and to authorized health care professionals for the evaluation of disease activity and of the effectiveness of treatments. Study duration is planned to be 15 years. Results will be analyzed periodically using means and standard deviations for continuous variables, and frequencies for categorical variables. Relations between time points, variables, patient and treatment characteristics will be evaluated in random effects repeated measures models.

**Discussion:**

The Dutch Multiple Sclerosis Study is characterized by online patient-driven inclusion; online data acquisition; the use of PROs; the optional monthly completion of short questionnaires; the interactive use of personal study data by patients and authorized health care professionals for self-monitoring, self-management and multidisciplinary care; the expected representativeness of the study sample; and a long-term time horizon. The study will provide valuable data on long-term disabilities, disabilities perceptions and HRQoL in MS patients in The Netherlands.

## Background

Multiple sclerosis (MS) is a chronic disease of the central nervous system, pathologically characterized by immune-mediated inflammation, demyelination and axonal degeneration. In most patients the initial disease course is characterised by relapses and remissions: relapsing remitting MS (RRMS) [1]. After a first episode patients may be diagnosed with Clinically Isolated Syndrome (CIS) suggestive of MS [1]. In CIS and RRMS immune activity and inflammation are the major underlying processes. Due to recurrent relapses and incomplete remissions RRMS patients often experience a step-wise increase in disability [1]. Since about two decades the number and severity of relapses can be reduced by treatment with immunomodulating or immunosuppressive disease modifying drugs (DMDs), as a result of which further increase in disability may be slowed or stopped [2, 3]. Long-term treatment with potent DMDs may even lead to an improvement of neurological functions [4].

After approximately 10–15 years most patients with RRMS convert to the Secondary Progressive (SP) phase, that is characterized by a slow and relentless increase in disability and a virtual absence of relapses [1]. DMDs have been reported to delay the conversion to SPMS and also to slow down the rate of progression once the SP phase has started [5–9]. In about 10-15 % of MS patients symptoms start and develop slowly without relapses: Primary Progressive MS (PPMS). In both SPMS and PPMS the continuous increase in disability is thought to result from degenerative processes and the disease course cannot be modified by treatment with the available DMDs.

MS profoundly diminishes health-related quality of life (HRQoL) due to the fact that symptoms often interfere with physical, cognitive, social or occupational activities [10]. A primary determinant of impaired HRQoL in MS is fatigue [11], occurring in over 80 % of patients [12]. It has been reported that DMD treatment may improve MS-related fatigue, but long-lasting benefits have not been documented [13].

Prospective long-term studies on disabilities and HRQoL in treated and untreated MS and CIS patients in real life settings are scarce. Such studies have been performed in the U.S.A., Canada, France and Italy [6, 7, 14–18], but they either date from the pre-DMD era, were restricted to specific regions or mainly involved patients treated in academic centres. Consequently, the findings are not applicable to the present MS population or cannot readily be generalized to patients in other countries or to those treated in general practices. As far as Dutch studies are concerned, Zwanikken in his thesis provided a detailed evaluation of the epidemiology, disability and HRQoL in Dutch MS patients [19]. However, these data were obtained in the era before the availability of DMDs and in the northern provinces only. To our knowledge recent data on long-term clinical changes in MS patients are not available in The Netherlands.

In a study on the costs of MS in the Netherlands it was found that the total mean costs per patient are driven essentially by the severity level, increasing from euro 9,300 per year at Expanded Disability Status Scale (EDSS) scores of 0–1 to euro 78,000 per year at EDSS scores of 8–9 [20]. The costs related to a relapse vary from approximately euro 50 to euro 9,000, depending on the relapse intensity [21]. Against this background of economic consequences of MS, it is important to be informed about the long-term changes in disability levels in MS patients [22]. Moreover, given the budgets restrictions to the Dutch health care system, the allocation of resources for MS care, both on macro-, meso- and micro-levels, should conceivably also be guided by the priority given by patients themselves to the various symptoms. Thus, patients’ perception of disability-related problems may help health care professionals to prioritize their care according to the subjective relevance of disabilities. HRQoL is an overall measure of wellbeing from a patient’s perspective and, as a patient-reported outcome (PRO), it can easily be assessed without involvement of neurologist or MS nurse, and yet providing a comprehensive measure of health status [8–10]. HRQoL is increasingly becoming an important measure in the assessment of disease burden in MS and in the evaluation of the effectiveness of DMD treatment [23, 24].

In view of the above, the availability of prospectively acquired data about the long-term changes in disability, disability perception and HRQoL in MS patients in the Netherlands will entail various significant advantages. In RRMS patients, long-term data will enable the evaluation of the effectiveness and therapeutic value of DMD treatment and thus establish a new natural history of the disease [6]; in SPMS and PPMS patients, information on long-term changes provides a point of reference for the real-life effectiveness of future DMD treatments. Moreover, long-term data on disability perception and HRQoL could be relevant to the comprehensive assessment of present and future needs of MS patients in terms of multidisciplinary care. This information may also be useful for MS patient organisations, looking after the interests of their members, e.g. in their communications with health insurers, and to health authorities, seeking guidance for policy decisions. For these various reasons we conceived and started the Dutch Multiple Sclerosis Study.

## Methods/Design

### Study objectives

The primary study objectives are to assess in patients with MS the long-term changes in 1) disabilities, 2) disabilities perception and 3) HRQoL. The secondary objectives are to assess the interrelations between 1) changes in disabilities and changes in disabilities perceptions, 2) changes in disabilities and changes in HRQoL, 3) relapses and changes in disabilities, 4) (adherence to) DMD treatment and changes in disabilities, and 5) (adherence to) DMD treatment and change in fatigue.

### Study design

The Dutch Multiple Sclerosis Study is a prospective, web-based, patient-centred, longitudinal, observational study in The Netherlands, with patient-driven inclusion. Given the innovative character of the study concept initially a follow-up of 2 years was planned to assess the feasibility of the project. Since then the study has been extended annually and is now expected to have a follow-up of 15 years.

### Study population

The population under study are the MS and CIS patients in The Netherlands. Accordingly, the eligibility criteria for patients to participate were not restrictive: 1) diagnosis of MS or CIS, 2) willing and able to comply with the study protocol, 3) having access to the internet, and 4) having given informed consent.

### Patient recruitment

Patients were informed on the possibility to participate via the websites of the patient organisations National MS Foundation The Netherlands (NMSF) (www.nmsf.nl) and Multiple Sclerose Vereniging Nederland (MSVN) (www.msweb.nl), the website of the MS4 Research Institute (www.ms4ri.nl), the study website www.msstudie.nl. By regular mail neurologists and MS-nurses in The Netherlands were sent an informative letter with patient brochures, which they were asked to hand out to their patients. The brochure was also sent to the patrons of the NMSF as an attachment to the foundations’ quarterly journal and related mailings. In the journal, study information was presented by the principal investigator (PJJ). Twice information about the study was published in health specials of large national and regional Dutch newspapers.

The informed consent text was available on www.msstudie.nl en www.ms4ri.nl. For further information the principal investigator (PJJ) could be contacted via www.ms4ri.nl. Patients who were willing to participate confirmed that they had read the information and gave their informed consent online by clicking on a specific page of the study website. The electronic consent also pertained the storage of personal data and the responses in the database. Once informed consent was obtained, a patient could start the baseline assessment.

### Ethical aspects

The protocol was submitted to the ethics committee Medisch Ethische Toetsing Onderzoek Patiënten en Proefpersonen in Tilburg, The Netherlands (nr M379). The committee concluded that a review was not indicated, as the study did not qualify for being tested according to the Dutch Medical Research Involving Human Subjects Act of 1999 (http://wetten.overheid.nl/BWBR0009408) [25]. The study is being performed in agreement with the Declaration of Helsinki (Ethical Principles for Medical Research Involving Human Subjects version 2013; 64th World Medical Association General Assembly, Fortaleza, Brazil, October 2013) (www.wma.net) and the Wet medisch-wetenschappelijk onderzoek met mensen (www.wetten.overheid.nl/BWBR0009408). The data analyses plan will be checked by an independent supervisory board (Stichting Infometer). Patients were informed that they have the right to discontinue their participation or withdraw their consent at any time and are not obliged to state their reasons. The completion of the questionnaires takes about 30–45 min every six months; the optional completion of the monthly questionnaires takes about 15 min every month; every three years the assessment of the EDSS by phone takes about 10 to 20 min (see below).

### Data acquisition

After having given their consent, patients received a personal code and logged on to the website of the study www.msstudie.nl, to choose a username and password. Online they go through various web pages containing the electronic case record forms (e-CRFs) with questions and questionnaires. Patients are informed by e-mail that an assessment is due and that the corresponding e-CRFs have been made available for completion. E-CRFs are to be completed within one week. Within this time frame e-CRFs may be filled in at moments that are suitable to the patient. Completion may take as many sessions as needed, as answers are saved automatically. The items of the questionnaire are fixed and the responses are automatically captured. Automated completeness checks are done before questionnaires can be submitted. The respondents see an overview of all questions and answers before submission and they can change the answers before submitting. After confirmation the e-CRF is automatically sent to the study centre. After submission changes are no longer possible. In case a e-CRF has not been completed within one week the help desk sends a reminder by e-mail.

### Technical aspects

The study is a modular application on the Curavista e-Health Platform, built on an Oracle database with JAVA-scripting, XML-applets and AJAX protocols. Data processing is 256-bits encrypted with VPN-tunnelling. The databases are physically and software secured in a dedicated data centre in The Netherlands. The database of the study is compliant with EU-regulations on data storage and activation for medical purposes. There are four separated databases: one with personal identifiers (name, address, identification number), one with medical records (answers to the questions, identification number), one with the social security number, and one with the key. Only after login the data are presented as a whole on the screen (encrypted key).

### Outcome measures

#### Disabilities and disabilities perception

The EDSS is a widely used disability measure in MS. The EDSS quantifies disability in eight functional systems and allows neurologists to assign a functional system score in each of these [26]. The functional systems are: pyramidal, cerebellar, brainstem, sensory, bowel and bladder, visual, cerebral and other. EDSS steps 1.0 to 4.5 refer to patients with MS who are fully ambulatory, and EDSS steps 5.0 to 9.5 are defined by the impairment to ambulation. A version of the EDSS that can be used by phone is available and will be used in this study [27].

The MSIP is a measure of MS-related disabilities and perception of disabilities with established psychometric properties [28, 29]. The MSIP is based on the International Classification of Functioning, Disability and Health and reflects an objectified view of the prevalence and severity of the impact of MS. The MSIP comprises 36 questions assessing disability (Q1a-Q36a) and disability perception (Q1b-Q36b) in the domains Muscle and Movement Functions (MMF), Excretion and Reproductive Functions (ERF), Basic Movement Activities (BMA), Activities of Daily Living (ADL), Participation in Life Situations (PLS), Environmental Factors (EF), Mental Functions (MF), and the symptoms fatigue, pain, speech and vision. The MSIP yields validated domain scores, ranging from 0 to 12 (ERF, MF), 0 to 15 (BMA), 0 to 16 (MMF), 0 to 20 (EF), 0 to 24 (ADL), and 0 to 26 (PLS), and symptom scores, ranging from 0 to 4. Higher scores indicate a worse condition.

#### HRQoL

HRQoL is assessed with the MSQoL-54 and the LMSQoL questionnaires. The MSQoL-54 is a psychometrically validated MS-specific multi-dimensional inventory of patient-centered health status [13]. It consists of the 36-item Short Form health survey as a generic core measure to enable comparisons to other patient populations and to the general population, supplemented with 18 additional questions exploring items relevant to patients with MS in the areas of health distress (four items), sexual function (four items), satisfaction with sexual function (one item), overall quality of life (two items), cognitive function (four items), energy (one item), pain (one item) and social function (one item) [30]. The MSQoL-54 contains 52 items distributed into 12 scales, and two single items. A physical and a mental dimension underlie the MSQoL-54: the physical and mental domains. Scores for each domain range from 0 to 100, where higher values indicate better HRQoL.

The Leeds Multiple Sclerosis Quality of Life (LMSQoL) questionnaire is a psychometrically validated scale that consists of eight questions, examining MS-related aspects of QoL over the past month [31]. Answers are rated on a 5-point scale from 0 to 4. The resulting score ranges from 8 to 32, with higher scores reflecting higher levels of well being. In a study of MS patients with acute relapses, it was found to be responsive to change with higher effect sizes than the sub-scales of the MSQoL-54, and it also showed a correlation with a detailed impact diary [32].

#### Relapses

Patients report the occurrence of relapses, the relapse intensity and eventual steroid treatment over the past six months.

#### Medication and adherence to DMD

The Medication and Adherence Inventory gives a patient-reported update of medications that are taken, the number of missed doses DMD in the past month (if applicable), and the date and reason of DMD discontinuation (if applicable).

#### Fatigue

Fatigue is measured by the Multiple Sclerosis Fatigue Impact Scale 5-item version (MFIS-5), a validated, short questionnaire examining a patient’s perceived impact of fatigue on a variety of daily activities over the past month [33]. Answers to each question are rated on a 5-point scale from 0 to 4. The MFIS-5 total score consists of the sum of the raw scores on these 5 items and ranges from 0 to 20, where higher scores indicate more experienced fatigue.

### Assessment schedule

At baseline the MSIP, MSQoL-54, Relapse Report, Medication and Adherence Inventory and MFIS-5 were completed, and the EDSS score was assessed by phone (MH). At follow-up the MSIP, MSQoL-54, Relapse Report, Medication and Adherence Inventory and MFIS-5 are completed every 6 months, and the EDSS score is assessed every 3 years. The monthly completion of MFIS-5, LMSQoL and Medication and Adherence Inventory is optional.

### Statistical analyses

The statistical analyses will be performed at the Department for Health Evidence, University Medical Centre Radboud Nijmegen, The Netherlands. Results will be described using means and standard deviations for continuous variables, and frequencies for categorical variables. Relations between time points, variables, patient and treatment characteristics will be evaluated in random effects repeated measures models. Multi-variate analyses will be performed to examine the interrelations described under the section Ojectives.

### Study status

A total of 391 patients have been included, the first patient in May 2011 and the last patient in September 2012.

### Organisation and funding

The study is an initiative of MS4 Research Institute (PJJ), and carried out by the institute in collaboration with Curavista bv. The study’s Advisory Committee includes Prof. Raymond Hupperts, neurologist, Academic MS Centre Limburg, Sittard, The Netherlands, Dr. Freek Verheul, neurologist, Groene Hart Hospital Gouda, Gouda, The Netherlands, Dr. Thea Heersema, University Medical Centre Groningen, Groningen, The Netherlands, and Dr. Ruud van der Kruijk, neurologist, Slingeland Hospital Doetinchem, Doetinchem, The Netherlands.

The study is funded by the National MS Foundation The Netherlands, Curavista bv and MS4 Research Institute.

## Discussion

The Dutch Multiple Sclerosis Study is a prospective, web-based, patient-centred study of long-term disability, disability perception and HRQol in patients with MS in The Netherlands. The study has several distinguishing features. First, the web-based character: potential participants were informed via a downloadable patient information form, applied for participation via the study website, and approved online the informed consent form. Second, the study is patient-centred with a patient-driven inclusion, as all outcomes are PROs and patients included themselves online, whereas usually studies focus on doctor-reported data, and neurologists and nurses are instrumental in the selection and inclusion of patients. In fact, we established a research partnership with patients without formal selection by health care professionals. As to the PROs, given the intrinsic clinical relevance of PRO-based conclusions, the implementation of the study results into clinical practice or their utilization for policy decisions is without conceptual barriers. Third, the study is interactive in that the personal study data are made available online to the patient in tables and graphs and can be used for self-monitoring purposes. Patients may also authorize their neurologist, MS-nurse and other health care professionals online access to the completed questionnaires and scores. This information can be used by the patient’s caregivers. Thus, the MSIP provides a comprehensive overview of disabilities and disabilities perceptions, and the visual representation of the scores with colour codes facilitates the detection of worsening compared to the last visit; the MSQoL-54 score may help in the assessment of clinical disease activity from the patient’s perspective and in the evaluation of DMD treatment. The optional monthly use of the MFIS-5, LMSQoL and Medication and Adherence Inventory may contribute to the self-management of fatigue, and to the professional evaluation of symptomatic treatments. Notably, in those neurological practices where the standard performance of EDSS scoring is not feasible, the PROs generated in the study may provide valuable information. In all, it is thought that the interactive use of study data may improve the efficiency and quality of professional multidisciplinary care and facilitate self-management. The feedback of completed questionnaires, scores and inventories, and their utilization by health care professionals and patients (self-monitoring, self-management) is believed to add practical value to the study participation and may constitute an incentive to continue participation. Especially in long-term studies the prevention of drop-outs is important. Fourth, as patients were informed all over the country, irrespective of their place of residency or treatment setting (general neurologist or MS-specialised neurologist; academic centre or general hospital), the study population is likely to be representative of the MS patients living in the Netherlands. This will increase the external validity of the study results.

## Conclusions

The Dutch Multiple Sclerosis Study is a prospective, web-based, patient-centred, interactive, study of long-term disabilities, disabilities perception and HRQoL in patients with MS in the Netherlands. The innovative study design is characterized by online patient-driven inclusion; online data acquisition; the use of PROs; the optional frequent completion of short questionnaires; the interactive use of personal study data by patients and authorized health care professionals for self-monitoring, self-management and multidisciplinary care; the expected representativeness of the study sample; and the long-term time horizon. The study will provide valuable reference data on long-term disabilities, disabilities perceptions and HRQoL in MS patients in The Netherlands.

## Abbreviations

ADL: Activities of Daily Living
AJAX: Asynchronous JavaScript And Extensible markup language
BMA: Basic Movement Activities
CIS: Clinically Isolated Syndrome
DMD: Disease Modifying Drug
E-CRF: Electronic Case Record Form
EDSS: Expanded Disability Status Scale
EF: Environmental Factors
ERF: Excretion and Reproductive Functions
EU: European Union
HRQoL: Health-Related Quality of Life
LMSQoL: Leeds Multiple Sclerosis Quality of Life
MF: Mental Functions
MFIS-5: Modified Fatigue Impact Scale-5 items
MMF: Muscle and Movement Functions
MS: Multiple Sclerosis
MSIP: Multiple Sclerosis Impact Profile
MSQoL-54: Multiple Sclerosis Quality of Life-54 items
MSVN: Multiple Sclerose Vereniging Nederland
NMSF: National Multiple Sclerosis Foundation The Netherlands
PLS: Participation in Life Situations
PP: Primary Progressive
PRO: Patient-Reported Outcome
RR: Relapsing Remitting
SP: Secondary Progressive
VPN: Virtual Private Network
XML: Extensible Markup Language
